# Influence of a Thiolate Chemical Layer on GaAs (100) Biofunctionalization: An Original Approach Coupling Atomic Force Microscopy and Mass Spectrometry Methods

**DOI:** 10.3390/ma6114946

**Published:** 2013-10-25

**Authors:** Alex Bienaime, Therese Leblois, Nicolas Gremaud, Maxime-Jean Chaudon, Marven El Osta, Delphine Pecqueur, Patrick Ducoroy, Celine Elie-Caille

**Affiliations:** 1 MicroNanoSciences and Systems Department, Franche-Comté Electronique, Mécanique, Thermique et Optique—Sciences et Technologies (FEMTO-ST) Institute, 32 avenue de l’Observatoire, 25044 Besançon Cedex, France; E-Mails: alex.bienaime@femto-st.fr (A.B.); therese.leblois@femto-st.fr (T.L.); nicolas_gremaud@yahoo.fr (N.G.); mj.chaudon@femto-st.fr (M.-J.C.); 2 Clinical and Innovation Proteomic Platform, University of Burgundy, CHU, 21000 Dijon, France; E-Mails: marven.elosta@clipproteomic.fr (M.E.O.); delphine.pecqueur@clipproteomic.fr (D.P.); patrick.ducoroy@clipproteomic.fr (P.D.)

**Keywords:** GaAs, self-assembled thiolate monolayers, proteins grafting, AFM, MALDI-TOF MS

## Abstract

Widely used in microelectronics and optoelectronics; Gallium Arsenide (GaAs) is a III-V crystal with several interesting properties for microsystem and biosensor applications. Among these; its piezoelectric properties and the ability to directly biofunctionalize the bare surface, offer an opportunity to combine a highly sensitive transducer with a specific bio-interface; which are the two essential parts of a biosensor. To optimize the biorecognition part; it is necessary to control protein coverage and the binding affinity of the protein layer on the GaAs surface. In this paper; we investigate the potential of a specific chemical interface composed of thiolate molecules with different chain lengths; possessing hydroxyl (MUDO; for 11-mercapto-1-undecanol (HS(CH_2_)_11_OH)) or carboxyl (MHDA; for mercaptohexadecanoic acid (HS(CH_2_)_15_CO_2_H)) end groups; to reconstitute a dense and homogeneous albumin (Rat Serum Albumin; RSA) protein layer on the GaAs (100) surface. The protein monolayer formation and the covalent binding existing between RSA proteins and carboxyl end groups were characterized by atomic force microscopy (AFM) analysis. Characterization in terms of topography; protein layer thickness and stability lead us to propose the 10% MHDA/MUDO interface as the optimal chemical layer to efficiently graft proteins. This analysis was coupled with *in situ* MALDI-TOF mass spectrometry measurements; which proved the presence of a dense and uniform grafted protein layer on the 10% MHDA/MUDO interface. We show in this study that a critical number of carboxylic docking sites (10%) is required to obtain homogeneous and dense protein coverage on GaAs. Such a protein bio-interface is of fundamental importance to ensure a highly specific and sensitive biosensor.

## 1. Introduction

In the field of biosensors, both the transducer and the bio-specific interface are considered as the cornerstones of each device. The performances of the sensors are conditioned to control the building of the interface, which requires physical processes, chemical and biochemical functionalization steps and biorecognition events in complex biological samples. The substrate plays a key role in this regard. It combines the possibility to graft biological elements onto a surface and to detect on it specific events thanks to the transducer properties. Some typical examples are gold for surface plasmon resonance (SPR) [1] and quartz for surface acoustic wave devices [2].

Among active materials, Gallium arsenide (GaAs) is of particular interest for many reasons. Firstly, GaAs is a crystal that presents interesting physical properties, especially piezoelectric and piezoresistive effects, which are used for sensor transducing. Secondly, the microfabrication processes are well known, making it possible to miniaturize components and to develop a specific interface. In this way, we designed an original piezoelectric resonant biosensor based on lateral field excitation that generates bulk acoustic waves. This original design, presenting the electronic part separated from the biological interaction reactor, makes it possible to detect a very low mass variation of captured peptides on the surface [3]. This promising sensor structure is obtained using wet etching, which is a low-cost and reproducible process that gives a specific behavior to the surface [4] thanks to adapted microstructuration. Moreover, without the requirement of an added layer [5,6], direct protein grafting is possible through a specific interaction between crystalline facets and specific amino-acid sequences [7,8,9,10,11,12], or genetically engineered proteins [13]. Another method consists of using an intermediate chemical self-assembled monolayer [14,15] (SAM), which offers the opportunity to control the orientation of a large choice of native proteins. Moreover, this “self-assembled-based process” has some advantages, namely it is easy, fast and inexpensive.

For SAM chemistry, the possibility to use either the bare substrate or the native oxidized layer offers a large choice of chemical interfaces for biofunctionalization. In particular, alkanethiol-based SAMs on III-V semiconductors are extensively used. The precursors in this field are probably Lunt *et al*. [16] and Nakagawa *et al*. [17], but their works were performed with a view to conferring specific functionalities on the surface through the modification of chemical and electrical properties of the material [18,19,20]. The most widely studied self-assembled monolayer is the octadecanethiolates monolayer [18], but various molecules could also be adsorbed onto the bare substrate thanks to their sulfhydryl groups. Among them, different types of variable-length chains (alkane, PEG, biphenyl *etc*.) have been used and terminal tail groups adapted (–CH_3_, –OH, –COOH, –NH, –SH *etc*.) according to the application being envisaged [21]. For biosensor applications, different strategies could be adapted [15] as seen in Dubowski* et al.* [22] and Adlkofer* et al.* [23]. These authors give us some examples of functionalization of specific chemical interfaces adapted for biological applications.

In this paper, we developed a specific chemistry composed of mixed thiolate molecules with hydroxyl and carboxyl tail groups [24] on GaAs crystals, developed originally on gold substrates for SPR measurements. This mixed layer is well known to make it possible, on the one hand, to covalently bind proteins to the material surface thanks to the activation of the carboxylate groups with amine-reactive esters [25]; and on the other hand, to reduce non-specific adsorption, thanks to the presence of inert hydroxyl tail groups. Furthermore, different chain lengths between these two molecules are used to render the –COOH tail group more easily accessible for protein grafting [20,26,27]. It was observed by several authors that a 100% monolayer of MHDA thiolates gave bad results in terms of protein graftings, especially due to heterogeneity and imperfections in the layer: repulsions between COOH groups creating sort of “holes” in the layer, interactions between COOH groups and certain substrates. Mixing the MHDA thiolates with shorter thiolates (like MUDO here) revealed to be conducive to a better MHDA orientation, repartition and efficiency on a surface. The possibility to use these molecules to functionalize the GaAs surface has previously been shown [28,29,30]. We optimized and analyzed the covalent grafting of proteins on this chemical interface thanks to an original characterization. The methods we used were atomic force microscopy (AFM) and matrix-assisted laser desorption/ionization time-of-flight mass spectrometry (MALDI-TOF MS). We wanted to engage this characterization study on several different thiolate layers reconstituted on the GaAs surface. This means that we had to use relatively high quantities of proteins. Albumin is one of the less expensive proteins, that is the reason why we chose it. Our results give comfort to what we could expect with an antibody monolayer, that would certainly give less non-specific interactions with the surface and then even more pronounced specific results. In this work, different ratios of hydroxyl and carboxyl end groups were tested to highlight their influence on protein grafting.

## 2. Results and Discussion

### 2.1. Characterization of the Presence of a Mixed Thiol Layer on GaAs Surface

**Figure 1:**
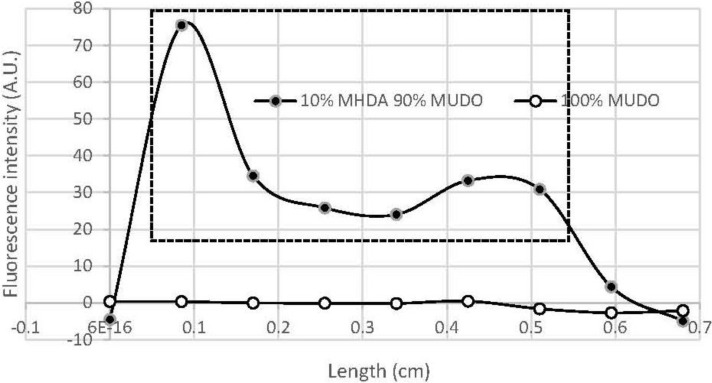
GaAs functionalized surfaces after incubation with Cy5-NHS fluorophore. GaAs samples functionalized by 10% mercaptohexadecanoic acid (MHDA)/90% 11-mercapto-1-undecanol (MUDO) (black dots) or 0% MHDA/100% MUDO (transparent dots). Fluorescence intensity emission at 680 nm is given in arbitrary units, while exactly the same conditions of excitation and filters were used in both cases of functionalization. The fluorescence was registered on the GaAs sample along a straight line, crossing the area functionalized by cy5-NHS (the cy5-NHS drop covers roughly 1 cm^2^ of the GaAs sample—the fluorescence measurements gave the results in the window that is delimited by the dashed line on the graph).

Figure 1 presents the fluorescence intensity obtained on the GaAs surfaces, functionalized by 10% MHDA/90% MUDO or 0% MHDA/100% MUDO.

Through this characterization, we managed to obtain a Cy5-NHS fluorescence response on functionalized GaAs samples. The fluorescence on 10% MHDA is not uniform along the whole line (0–0.7 cm), simply because the GaAs sample was not entirely covered by the NHS-fluorescent probe during the graftings. Only one part of the GaAs functionalized surface (roughly 1 cm^2^) was covered by the NHS fluorescence probe. This allows us to have a control area on the same sample just next to the area covered by the probe. Thus, the fluorescence proves 1) the presence of COOH groups on the mixed thiolate layer, and 2) the binding specificity of the NHS-molecule (here cy5-NHS, and then EDC-NHS used for protein grafting in the following step) to the MHDA/MUDO functionalized GaAs surface.

### 2.2. Chemisorption of Mixed Thiol Layer on GaAs Surface

**Figure 2:**
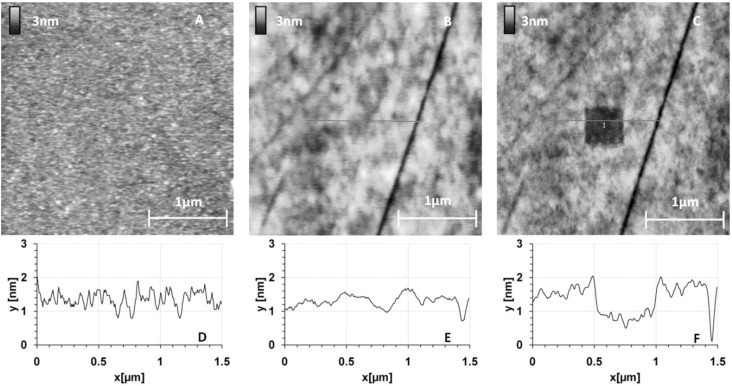
GaAs surface before (**A**) and after (**B**,**C**) thiolate functionalization (10% MHDA) and their corresponding sections (respectively **D**,**E**,**F**).

Before protein grafting, we checked the organization of a 10% MHDA layer on the GaAs (100) surface. Figure 2 shows the surface topography of this layer.

Figure 2A shows the initial surface state of a bare GaAs wafer. Figure 2B,C correspond to the same functionalized GaAs surface, without and with a 500 nm scraped zone respectively. Images of cleaned and functionalized surfaces show a very low roughness (z range 3 nm) corresponding to a highly smooth surface similar to those presented in other publications [8,23,31,32,33]. Since the GaAs sample is polished on one side (see experimental section), topography images can reveal the presence of stripes on the substrate. These stripes give the black lines on the AFM images in Figure 2B,C).

The functionalized surface is even smoother (Figure 2E), which is confirmed by the decrease in arithmetic average and root mean square roughness values (Ra and RMS respectively), from Ra = 0.23 nm/RMS = 0.32 nm for a naked surface to Ra = 0.18 nm/RMS = 0.23 nm for a chemically functionalized surface. To measure the thickness of this layer, we made a hole by scraping a square of 500 nm width with a stiff cantilever (k = 0.58 N/m) (Figure 2C). This scraping makes it possible to locally remove the layer of thiols as seen by a well-defined dark square. The section profile (Figure 2F) highlights the material removal at this place and we observed a layer thickness of approximately 1 nm. Although this method precludes precise estimation of the thickness of the mixed thiol layer and any conclusion regarding its organization or composition, the AFM results tend to demonstrate that a homogeneous mixed thiolate layer has been established. The obtained thickness suggests that this layer is organized as a monolayer because this value is in the same range as those found by Zhou and Walker [34] with single wavelength ellipsometry (SWE) measurements: 1.7 nm for MHDA and 1.4 nm for MUA (for 11-mercaptoundecanoic acid, a thiolate composed of 11 carbons, terminating with a carboxyl tail group). Then, we used complementary characterization methods like contact angle, ellipsometry and X-ray photoelectron spectroscopy (XPS) to validate the good organization and composition of this chemical layer. These results have previously been published [28].

### 2.3. Protein Grafting on the Functionalized GaAs Surface

**Figure 3:**
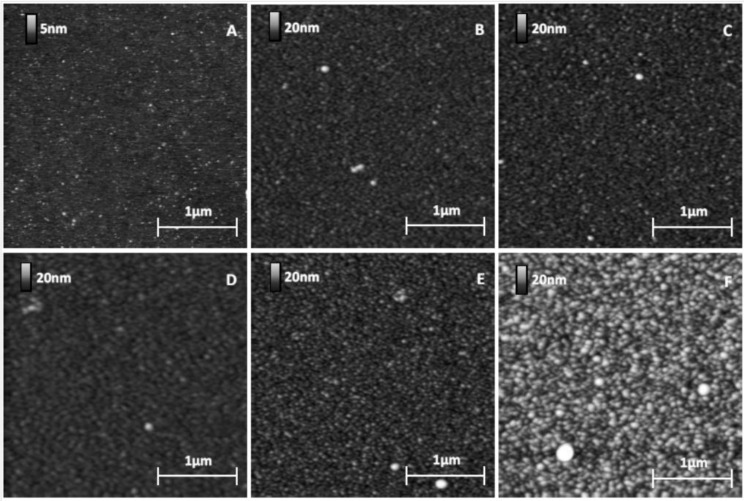
GaAs surface images before (**A**) and after protein grafting for several chemical interfaces: unfunctionalized (**B**); 0% MHDA (**C**); 100% MHDA (**D**); 3% MHDA (**E**); and 10% MHDA (**F**).

The protein was covalently grafted to MHDA / MUDO mixed SAM through its amine groups using the EDC/NHS activation of COOH docking sites presented by MHDA molecules. After the incubation of this surface with proteins, followed by a washing step with a 40 mM Octyl Glucoside solution, carboxyl group deactivation was finally performed with ethanolamine. The protein covered surfaces (Figure 3D–F) and some references surfaces (Figure 3A–C) were characterized.

**Figure 4:**
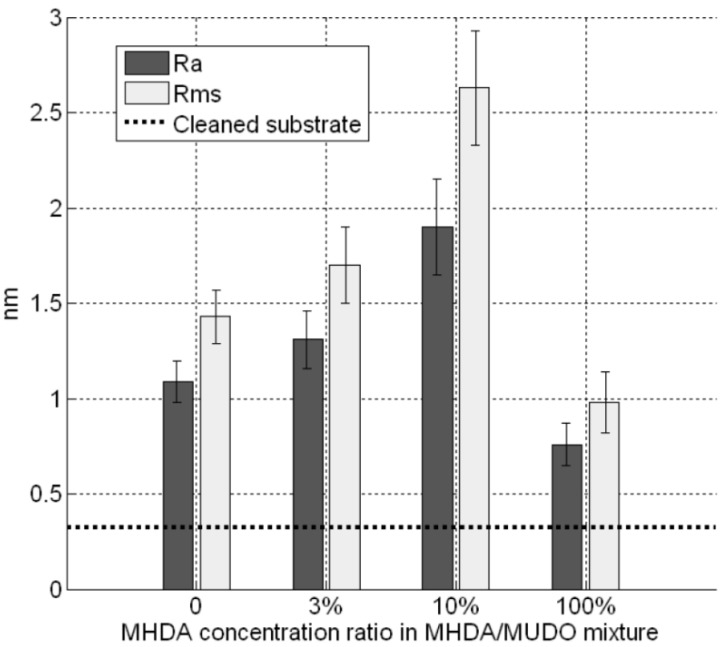
GaAs surface roughness after protein incubation on different chemical interfaces.

The images (Figure 3) give results on different chemically modified GaAs surfaces in the same experimental imaging conditions, as regards AFM tips and scan settings. Repeatability and reliability of results were controlled: three samples for each condition were taken, and measurements were performed in different places on each sample. Compared to the reference sample (Figure 3A), we clearly observed a modification of the GaAs surface after the protein grafting protocol on the different chemically modified substrates. The initial very smooth surface (z range of 5 nm) was modified and the roughness strongly increased after this step (z range of 20 nm for the other samples). Figure 4 gives RMS values as a function of the MHDA percentage. The unfunctionalized (Figure 3B) and 0% MHDA (Figure 3C) surfaces behaved similarly, presenting fine and small grains. These motifs are probably proteins or aggregates of proteins. In spite of the detergent washing, some proteins or aggregates remained absorbed on the surface. Nevertheless, the substitution of some MUDO by MHDA molecules induced a change on the surface topography and bigger motifs were observed in these cases. With the mechanical filtering induced by the tip, the observed motifs had a size between 5 and 15 nm, which is consistent with 3–8 nm RSA protein dimensions [35]. The 10% MHDA sample shows a highly rough surface compared to the others (RMS = 2.63 nm) and grafted proteins form a dense layer on the GaAs surface. The 3% MHDA covered surface presents an intermediate value of RMS. The 100% MHDA surface shows the least roughness. In this last case, it may be that the presence of supernumerary docking sites (100% MHDA) induced RSA grafting by several sites. These multiple protein-surface bonds could tend to flatten the proteins on the surface, thus smoothing out the surface (RMS = 0.98 nm).

The corresponding interpretation of these roughness values may be due to the number of docking sites offered by the different SAM layers. Indeed, if we assume the protein diameter to be 5 nm, a complete and saturated protein monolayer would give coverage of around 50,000 proteins per µm^2^. The density of the thiolate layer is approximately 5 × 10^6 ^molecules per µm^2^, because the surface occupied by a thiol on the GaAs (100) surface is roughly 20 Å^2^ [18,36]. The surface ratio of thiolates to protein would be 100 thiolates for one protein. Theoretically, with this complementary information, self-assembled monolayers composed of 3%, 10% or 100% MHDA should offer successively 3, 10 and 100 docking sites for one protein. At this stage, we have to keep in mind that the ratio of thiolates in the solution is certainly not respected on the surface, due to several parameters like the thiolate functional groups and their chain length. Moreover, if we consider that MHDA molecules are non-uniformly distributed and that only a part of the carboxyl groups reacts (only 10% of docking sites activated for Ding* et al.* [37]), we can assume that self-assembled monolayers are composed of an insufficient number of docking sites at 3% MHDA, an excess at 100% MHDA and a good compromise at 10% MHDA. Based on the AFM images, the 10% MHDA layer seems to be an ideal ratio to immobilize enough proteins to create a dense protein layer. Similar to our AFM images of RSA grafted on 10% MHDA surface, Duplan* et al.* [32] showed AFM images of immobilized neutravidin (molecules close to RSA in size, with a molecular weight of 60,000 Da,* versus* 64,500 Da for RSA) on GaAs modified by polyethylene glycol mixed thiol, and confirmed the ability to form an organized, densely packed protein layer on the GaAs surface. The authors used a ratio of 1/15 (6.66%) biotinylated docking sites distributed in a hydroxyl terminated PEG layer.

### 2.4. Protein Covalent Binding or Physisorption?

**Figure 5:**
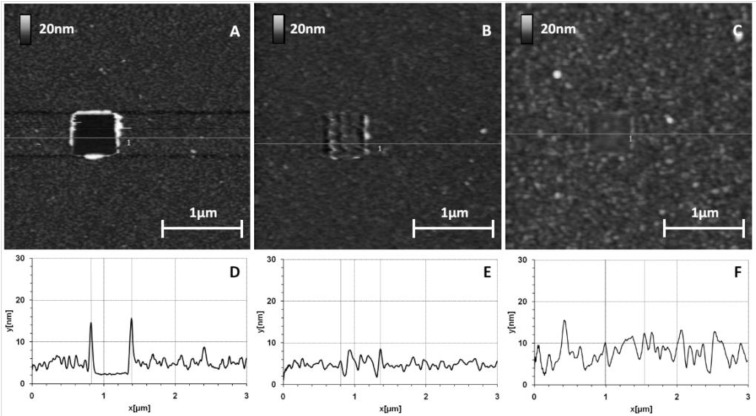
Atomic force microscopy (AFM) images of medium scrapings (stiff tip k = 0.12 N/m) applied on GaAs bio-functionalized surfaces and corresponding sections: 0% MHDA (**A**,**D**); 100% MHDA (**B**,**E**); and 10% MHDA (**C**,**F**).

Figure 5 shows surface modifications after the protein deposition step, although the cleaned bare and 0% MHDA functionalized surfaces do not present functional chemical groups able to fix protein. To highlight the binding properties between the protein and each chemically modified surface, we tried to scratch the protein and thiolate layers by applying a controlled contact force with the tip on the surface. As explained in the experimental section, various AFM tips were used to apply medium or strong scraping tests. The applied force on the sample was adjusted by varying the contact setpoint and the same settings were used for each sample. A first set of tests was performed by applying a moderate force on the surface (scraping zone of 500 nm by 500 nm) thanks to a medium stiff tip (NPS10-B, k = 0.12 N/m). Results are reported in Figure 5.

We observed that:

(i) The 0% MHDA and bare substrate surface (not presented) have the same typical physisorption behavior when incubated with the protein solution. The gallium arsenide surface is known to be attractive for protein adsorption but in our experiments, the GaAs side was relatively poorly covered after protein incubation, probably due to the efficiency of OG washing, as shown previously by Ding* et al.* [37]. On AFM images obtained on naked GaAs surfaces (data not shown), the step between the protein layer and the substrate was almost invisible and the rolls were very small. The GaAs naked surface does not seem to adsorb a lot of RSA protein.

(ii) On MUDO functionalized GaAs surfaces, the deposited proteins were easily scratched by this test and a square zone 500 nm wide and 3.5 nm thick appeared. On the sides of this square, we observed rolls corresponding to a heap of proteins displaced by the tip. The visible rolls prove that proteins are present on MUDO surfaces, but only weakly adsorbed since they are easily removed from the surface by a medium scraping test.

(iii) On the contrary, the 100% MHDA surface is not fundamentally affected by this scraping test. In the scraped zone, the surface appears “compacted” and the molecules appear to be stretched but not removed under the force applied by the tip. As the consequences of the scraping were limited to the apparent spreading (without pulling off) of molecules, it tends to prove that proteins are strongly attached to the GaAs substrate.

The mixed layer containing 10% MHDA behaved similarly to 100% MHDA: the surface on the scraped zone appeared “compacted” with molecules that stayed on the surface while applying moderate force. Surprisingly, only 10% MHDA thus seems sufficient to toggle to a strongly bound protein layer. This result highlights the covalent binding that exists between the substrate and the protein, when MHDA molecules constitute the chemical interface.

**Figure 6:**
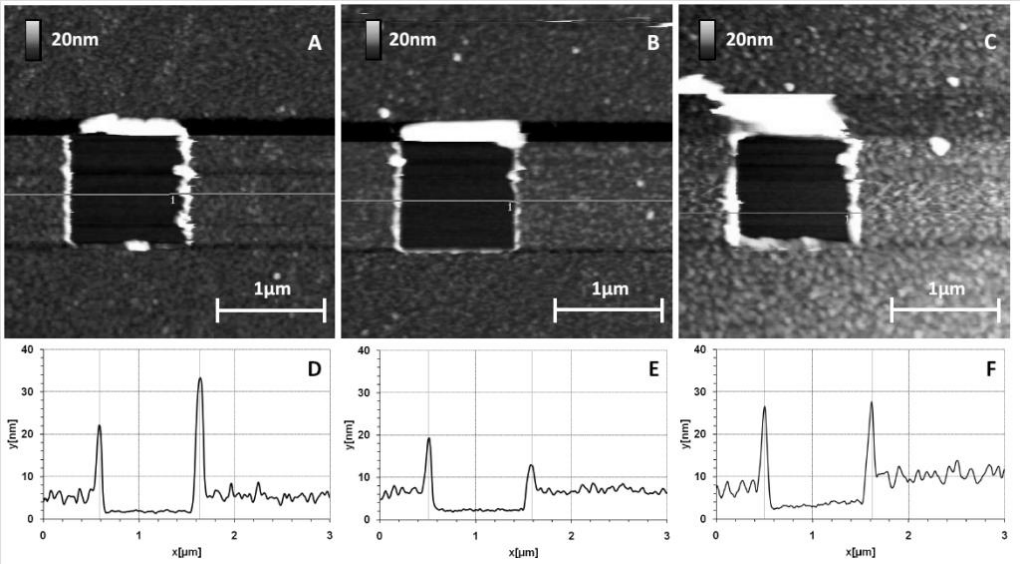
AFM images after strong scraping applied to GaAs biofunctionalized surfaces. Images and corresponding sections: 0% MHDA (**A**,**D**); 100% MHDA (**B**,**E**); and 10% MHDA (**C**,**F**).

On the section of Figure 5D, corresponding to the 0% MHDA surface, we show the height of rolls due to the scraping. The considerable height (10–15 nm) is evidence of the material having been removed from the scraped zone. At the bottom of this 500 nm wide hole, we observed a very flat surface corresponding to the GaAs substrate, and we measured a protein layer thickness of 3.5 nm (see explanation below for strong scraping and Figure 6).

The protein adsorption observed on the 0% MHDA layer is surprising because the hydroxyl tail group of MUDO is known to limit non-specific adsorption compared to other tail groups, and the OG washing should have completely removed the protein on the bare substrate. Recent articles [18,29,38,39] provide a possible explanation for this phenomenon. The length of the MUDO alkane chain, composed of 11 carbon atoms, is relatively short and it seems that a chain of 15 or 16 carbon atoms is the minimum chain length required to obtain a high degree of self-organization. This disorganization in the MUDO layer could then induce a sort of “porous” and loose chemical layer, in which aliphatic chains could interact with proteins through hydrophobic interactions. The addition of MHDA molecules (16 carbon atoms) in the functionalization process seems to facilitate the formation of a more densely packed protein layer, because no pulling-off at all was observed on mixed and 100% MHDA layers, as can be seen in Figure 5B,C.

### 2.5. Thickness of the Combined Thiol/Protein Layer

Additional scraping experiments were performed in order to totally remove the grafted protein layer and the thiol chemical interface. Similar experiments as in the previous section were performed, on a square area of 1 µm wide using the stiffest tip, an NPS10-A (*k* = 0.58 N/m). The force applied was considerably greater with this approach. The images and corresponding sections are presented in Figure 6.

**Figure 7:**
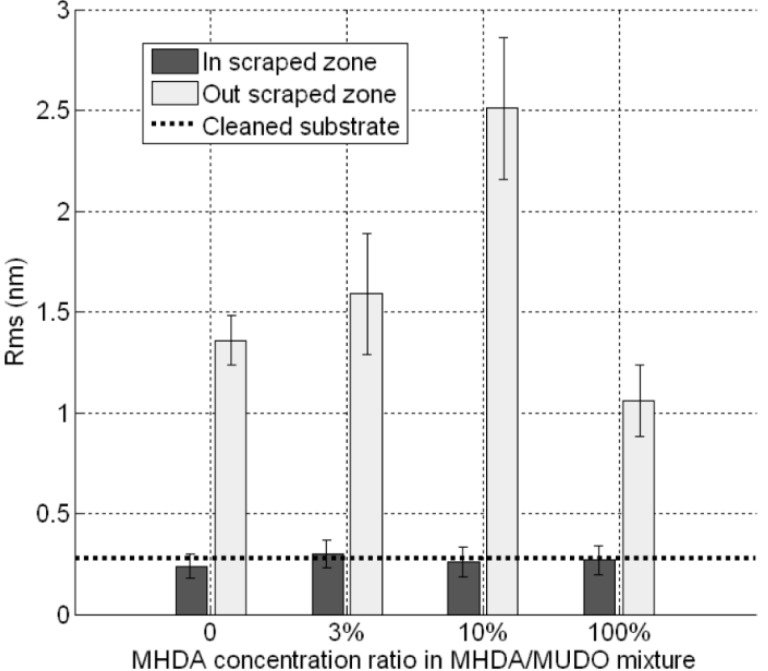
RMS values of protein modified GaAs surfaces, inside and outside the scraped zones, for each chemically modified GaAs interface.

For each surface, the thiolate/protein layers were scratched and we observed a well-defined square of 1µm wide corresponding to the scraped zone. For bare substrate (not presented) and 0% MHDA (Figure 6A), the images are identical to these obtained by the first medium-scraping test, supporting the idea that the protein layer is just adsorbed on the surface and is not strongly fixed. Applying a high force made it possible to remove materials from the surfaces composed of 100% MHDA (Figure 6B) and mixed interfaces (3% MHDA (data not shown), 10% MHDA (Figure 6C)). Large rolls were formed around the scraped zone, proving the displacement of proteins and probably of thiolate molecules. The volume of this roll makes it possible to establish a comparison of the quantity of scraped molecules. Cleaned bare substrate (not presented), 0% MHDA (Figure 6A), 100% MHDA (Figure 6B), 3% MHDA (not presented) and 10% MHDA (Figure 6C) show, in this order, the smallest to largest roll of scratched molecules. The corresponding sections of these images are presented in Figure 6D–F. In the scraping zone, we observe similar flat surfaces. In order to analyze the roughness of these flat surfaces, we performed cross-sections inside and outside the scraped zone. The corresponding RMS values are reported in Figure 7.

**Figure 8:**
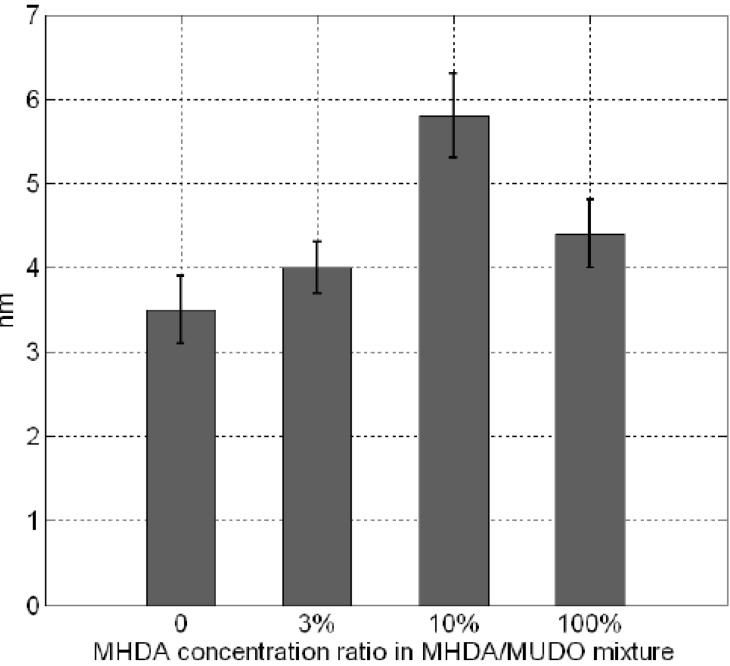
Thickness of the combined thiolate and protein layer, measured for each chemical interface.

As expected, outside the scraped zone, the RMS values are similar to these obtained in Figure 4. This result demonstrates the reproducibility of our GaAs biofunctionalization process. In the scraped zones, RMS values are approximately equal to 0.3 nm, which corresponds to the initial roughness of unfunctionalized substrate (dotted line). This result would indicate that, with the high force applied here, we scratched both thiolate and protein layers. Similarly, we measured the thickness of the bio-interface (Figure 8). The 0% MHDA profile (Figure 6D) is similar to Figure 5D and the average thickness of the layer is 3.5 nm (Figure 6D). This again highlights the reproducibility of our surface biofunctionalization process. The 100% MHDA sample presents a thickness of 4.4 nm (Figure 6E); mixed surfaces have a thickness of 4 nm and 5.8 nm for 3% MHDA and 10% MHDA (Figure 6F) respectively.

These thickness values could correspond to the superposition of the two layers: a 1.5 nm thin thiol layer [34] and the Rat Serum Albumin (RSA) protein layer [35]. The highest value, obtained at 10% MHDA, is in agreement with previous experiments, because the protein molecules do not tend to flatten on the surface due to the proximity of other neighboring RSA proteins. These results are in line with previous observations, namely:

The 0% MHDA surface allows protein adsorption.

The 100% MHDA surface presents a number of docking sites in excess of those required to obtain a protein monolayer. Indeed, on highly dense MHDA covered surfaces, proteins could graft to the surface, by engaging multiple free primary amine groups. The consequence of this could be the flattening of the protein on the surface.

The 3% MHDA surface is certainly limited in terms of number of docking sites, thereby reducing the density of protein coverage on this surface.

The 10% MHDA surface appears to be the best candidate, since this surface allows grafting of a dense, homogeneous and stable layer of proteins, and the number of docking sites appears to be well adapted for a biosensor interface.

### 2.6. *In Situ* MALDI-TOF MS Analysis on GaAs Surfaces Modified by Proteins: Identification and Quantitative Distribution

In parallel to AFM characterization of protein modified GaAs surfaces, we performed mass spectrometry analysis, in order to analyze the potential of the proteins present on GaAs to be ionized and identified. To this end, we split each GaAs sample into two parts to perform, firstly, AFM characterization, and secondly, mass spectrometry analysis. With these measurements, we aimed to investigate the correlation between the density of motifs observed by AFM and the MS signal intensity of the protein. The MS results yielded qualitative information regarding the protein layer coverage on the different chemically modified GaAs surfaces. Moreover, thanks to automatic measurements at different places on the whole surface, we were able to perform quantitative comparisons between each sample. This AFM/MALDI-MS coupled analysis is truly original, since, without the need for labeling, it provides complementary qualitative information beyond that obtained by photoluminescence [5,40] or labeled techniques like fluorescence [32,37,40].

The surface preparation, consisting of spraying TCEP, trypsin and HCCA matrix on the GaAs modified surfaces, was done on the whole surface, corresponding to a square area of 10 mm width. Afterwards, we screened the whole surface pitch by pitch with MS measurements to verify the homogeneity of protein coverage. Each pitch of this test is defined by a 120 µm wide square, composed of 2000 shots randomly distributed at steps of 20 µm. Among these measurements, we chose one position on each chip that showed the highest signal intensity. The protein generated trypsin fragments were matched to a database sequence. The protein having the closest peptide sequence was defined as the identified protein. A score, based on Mascot algorithm, was then attributed. This algorithm evaluates the probability that the identified protein is not a random match,* i.e.* that the identified protein is unambiguously the right one. In the same way, a Mascot score was attributed for MS^2^ analysis, to compare detected and theoretical fragmentations of a specific peptide (here the 1960 Da peptide). Table 1 summarizes these results.

**Table 1:** MS and MS^2^ results obtained on protein modified GaAs substrates.

MHDA concentration	MS			MS^2^ (peptide 1960 Da) Mascot score
	Identified protein	Matched peptides	Mascot score	
Bare substrate	–	–	–	–
0%	RSA	8	85.8	16.42
3%	RSA	8	66.9	21.78
10%	RSA	9	87	59.92
100%	RSA	10	101	17.68

The RSA protein was identified on each surface, except on bare GaAs substrate. The unfunctionalized sample spectra do not exhibit peaks, proving that RSA protein was not adsorbed on the bare GaAs surface. For other MHDA/MUDO surfaces, the mascot scores were significant for all GaAs substrates, which confirms that the motifs observed by AFM truly correspond to RSA proteins. Among eight peptides matched on 0% MHDA and 3% MHDA samples, only one peptide differed between samples. This could partly explain the lowest score of RSA identification on the 3% MHDA sample. For 10% MHDA, the sequence covered was the combination of the two spectra detected on the 0% MHDA and 3% MHDA samples, therefore increasing the score. The addition of the 983.6 Da matched peptide to this sequence resulted in the 100% MHDA surface having the highest score. The mascot score obtained and the number of matched peptides with GaAs substrates were close to the values obtained on a gold reference chip with the same biofunctionalization protocol.

**Figure 9:**
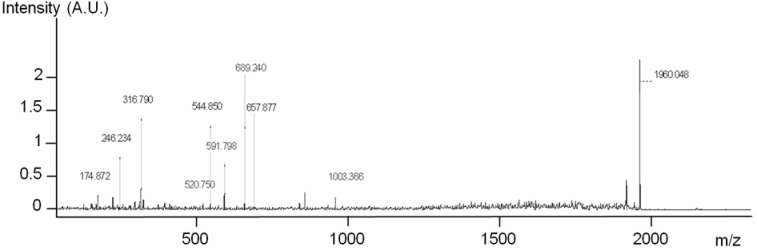
MS^2^ analysis of the 1960 Da peak on GaAs (100) functionalized with 10% MHDA after Rat Serum Albumin (RSA) grafting.

The MS^2^ measurements were performed on each functionalized chip. Mascot scores were calculated for the 1960 Da peptide, which is a specific peptide of RSA protein. A score of 60 was obtained on the 10% MHDA sample, but the score was around 20 for the other functionalized GaAs chips. The score obtained at 10% MHDA was the same as on the gold reference chip, providing unambiguous proof of the presence of the peptide 1960 Da on the surface, and highlighting that it is possible to investigate peptides* in situ* on the GaAs surface. The MS^2^ spectrum of this surface is presented in Figure 9.

**Figure 10:**
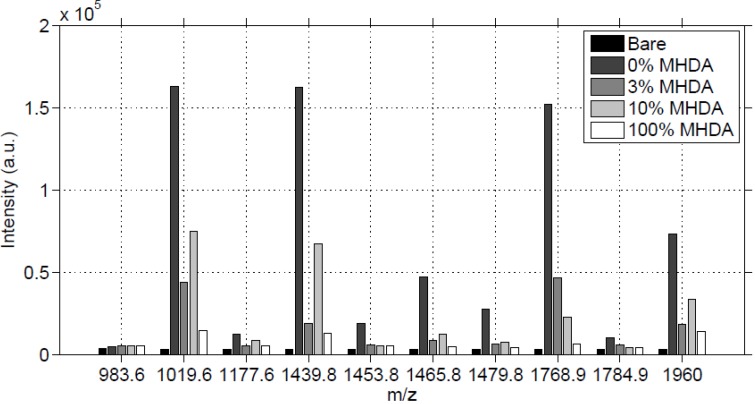
Sum of MS intensities for each matched peak, obtained on 2mm by 2mm unmodified (bare) and functionalized GaAs surfaces, after incubation in the RSA solution.

If the MS intensity of peak 1960 Da (Figure 10) reflected the amount of this peptide present on the surface, we would get the highest MS^2 ^score for 0% MHDA, a medium MS^2 ^score for 10% MHDA, and low MS^2 ^scores for 3% and 100% MHDA. Our results (Table 2, fifth column) seem to follow this pattern, except for the 0% MHDA surface, which gave the lowest score. This low value indicates that there are many species that are not attributed to this 1960 Da peak, giving us reason to think that this chemically modified surface induces perturbations in either protein fragmentation or peptide desorption, or even both.

Using the same experimental conditions, on a 2 mm by 2 mm defined area, we obtained semi-quantitative characterization of the protein interface on the GaAs surface, through the comparison of the matched peptide intensities. On each protein-modified GaAs surface, we collected a matrix of 17 by 17 MS spectra with the previous shooting conditions, and we summed the intensities of each matched peptide peak. The result is reported in Figure 10.

In Figure 10, for most of the MS peaks, we observed a high intensity for 0% MHDA, a medium intensity for 10% MHDA and low intensities for 3% and 100% MHDA. The bare substrate gives a noise reference and the low intensities of its peaks show once again that no RSA protein was adsorbed, proving the efficiency of the washing protocol. The highest intensity of the 0% MHDA surface is very surprising, meaning that this MUDO interface would authorize albumin non-specific interactions. Even after the OG washing step on this MUDO surface, proteins remained stuck on it. Probably the presence of hydroxyl terminal groups on MUDO enables the establishment of weak but numerous interactions with proteins.

**Figure 11:**
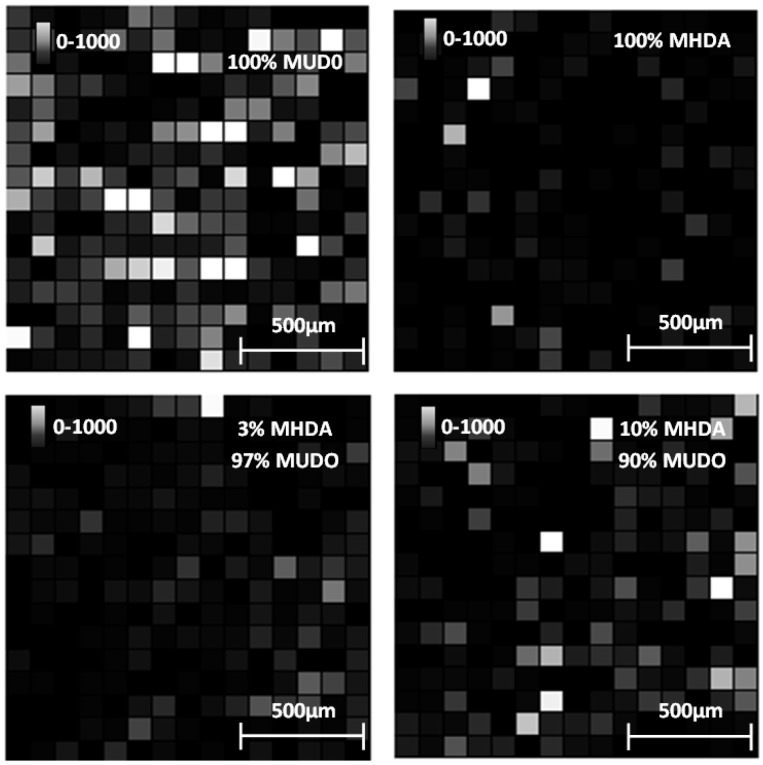
Intensity images (arbitrary unit) of the 1960 Da peak for each chemically functionalized GaAs surface after incubation in the RSA solution.

The intensities of surfaces covered with MHDA molecules show a gradation, from lowest to highest intensity, with MHDA concentrations of 100%, 3% and 10%. This gradation is expected, although it differs slightly from the AFM analysis. To illustrate these results, we report in Figure 11 the intensity of the 1960 Da peak for each position on the 4 mm^2^ tested surfaces.

We observe that the 0% MHDA surface (Figure 11) apparently presents the highest amount of RSA proteins. This observation differs from the AFM images, showing small and scattered RSA motifs, in comparison with MHDA surfaces. How can this difference between AFM and MS results be explained? It could be purported that peptide ionization is easier on the 100% MUDO (0% MHDA) interface than on the MHDA surface, and that more protein molecules are desorbed by the laser from the 100% MUDO surface. Peptide desorption could be facilitated by the disorganized MUDO thiolate monolayer, and by the lower interactions existing between the RSA protein and the chemical interface. In fact, as was observed during AFM scraping measurements, the link between protein and chemical interface is weak for 100% MUDO and strong for MHDA interfaces. Griesser* et al.* [41] proved that peptide MALDI ion signals decrease as the surface-peptide binding affinity increases, and that peptide ionization is very sensitive to the nature of the chemical interaction between the surface and the peptide [42]. This phenomenon is particularly true for whole protein detection. Although trypsin digestion strongly reduces this limitation, it may be possible that in our case, the different chemical interfaces play an important role during this process. The weak interaction between peptides and the alkane chain and the non-presence of carboxylic acids could facilitate molecule desorption, and generate a higher intensity of signal response.

This theory seems to be correct, in light of the results obtained on the 100% MHDA surface. The AFM images show that the protein layer is not significantly less dense than on 3% MHDA, but an over-abundance of docking sites that multi-bond RSA peptides could explain the low MS signal. For 3% and 10% mixed chemical interfaces, the MS results are less surprising, and are consistent with AFM observations: the more MHDA in the chemical layer, the more intense the MS signal. To determine the optimal number of docking sites necessary to obtain a well-organized and dense protein layer, we recently performed complementary analyses of surfaces containing percentages of MHDA molecules greater than 10 percent. These results are given in the following section.

### 2.7. Density of Carboxylic Docking Sites and Consequences on the Reconstituted RSA Protein Layer on (100) GaAs

In the previous results, we showed that, on the one hand, a concentration of less than 10% MHDA is not sufficient to graft a dense and homogeneous layer of proteins; and on the other hand, that a layer composed of 100% MHDA molecules induced multi-bonding of the proteins to the surface, which somehow flattened the proteins and reduced laser desorption of this protein layer. We investigated the behavior of the protein layer on chemical interfaces composed of more than 10% MHDA.

**Figure 12:**
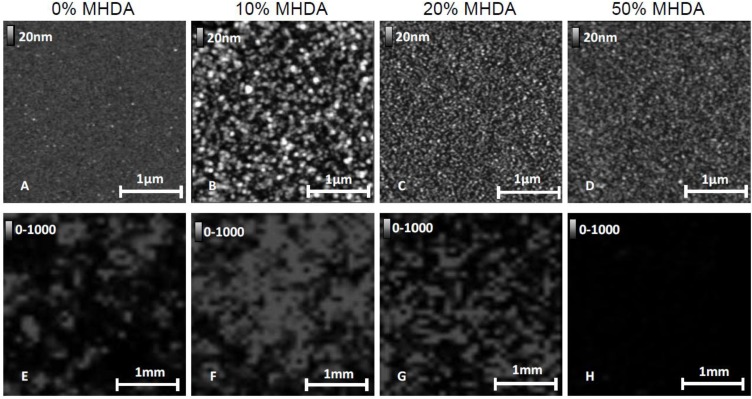
AFM images (**A**–**D**) and MS imaging results (**E**–**H**) for 0%–50% MHDA concentration: 0% MHDA (**A**,**E**); 10% MHDA (**B**,**F**); 20% MHDA (**C**,**G**); 50% MHDA (**D**,**H**).

We applied the same AFM and MS characterization methods as previously described to chemical layers obtained from a solution containing respectively 20% and 50% MHDA molecules. The AFM and MS images are reported in Figure 12.

Images A, B, E and F in Figure 12 give the same results as those previously observed for 0% MHDA (A,E) and 10% MHDA surfaces (B,F). On the 0% MHDA surface, we observed sparse patterns corresponding to RSA protein, and only locally high MS signal intensity. The 10% MHDA surface shows the highest organized protein layer and a strong MS signal. For highest values of MHDA (C,D,G,H), we observe a drastic decrease in the pattern sizes. The AFM images of these surfaces show the lowest patterns, whereas the MS signal intensity dramatically dropped and was even absent for 20% and 50% MHDA respectively. These results are in accordance with the hypothesis of multi-bonded proteins on such highly MHDA-concentrated surfaces. This observation suggests that a concentration of 10% MHDA apparently offers an optimal number of docking sites to graft a protein monolayer homogeneously and correctly.

## 3. Experimental Section

### 3.1. GaAs Samples

All GaAs samples were cut from a unique wafer provided by Azelis Electronics. This wafer was (100) oriented, semi-insulating and undoped. Only one side was polished, which avoids confusion during handling. We divided the wafer into several samples according to the advancement of the protocol using the (110) cleavage planes of the material to confirm that the initial surface was identical and that AFM and MALDI-TOF MS measurements were performed on the same original sample.

### 3.2. Functionalization of GaAs Samples

Samples were functionalized using a protocol previously described and characterized [28]. The first step consists of cleaning the surface using successively Decon^®^, acetone, sulfochromic acid (98% H_2_SO_4_, 2% K_2_Cr_2_O_7_) and ethanol. Samples were rinsed with deionized water and were dried with nitrogen gas between each step. Then, to deoxidize the GaAs surface, a HCl:H_2_O (1:1) solution was used. The reaction is automatically stopped (after 2 min at ambient temperature) because the acid solution only etches the oxide present on the surface revealing Ga and As atoms, which are necessary for thiolate functionalization (Ga–S and As–S bonds)[4]. Next, surfaces were functionalized by a self- assembled monolayer (SAM) of thiols. Two kinds of thiols were tested (Sigma-Aldrich^®^): mercaptohexadecanoic acid (HS(CH_2_)_15_CO_2_H, called MHDA) and 11-mercapto-1-undecanol (HS(CH_2_)_11_OH, called MUDO). Thiolate functionalization was achieved by immersion in solutions composed of 1 mM MHDA and/or MUDO diluted in ethanol, and incubation was carried out overnight at room temperature. Six solutions composed of *X*% of MHDA and *Y*% of MUDO, noted *X*% MHDA, were tested with *X* = {0, 3, 10, 20, 50, 100} and *Y* = 100 − *X*. After functionalization, protein grafting was performed. This protocol consists of:

The activation of carboxyl groups presented by MHDA molecules with *N*-hydroxysuccinimide (NHS) and *N*-(3-dimethylaminopropyl)-*N*-ethylcarbodiimide (EDC). (NHS coupling kit, Biacore^®^, 30 min), Rat serum albumin protein (RSA) grafting in an ultrasonic bath, prepared at 40 µg/mL in an acetate buffer solution at pH 5.2. (Sigma-Aldrich^®^, 20 min). Before any grafting of protein to a functionalized surface, the conditions for a better grafting have to be optimized. For that, we used to screen a range of pH values, usually from 4 to 7, for each protein, in order to determine the pH that allows the highest quantity of proteins to bind to the surface. Indeed, on MUDO/MHDA, after activation of COOH by EDC-NHS, the better pH for protein grafting is often a pH close to the isoelectric point of the protein. This pH is close to pH 5 for RSA protein.

Non-specific adsorption removed using Octyl β-*d*-glucopyranoside (OG) diluted in phosphate buffer saline solution (40 mM, PBS 1*x*, pH 7). (Sigma-Aldrich^®^, 10 min).

Passivation of GaAs surface with Ethanolamine (NHS coupling kit, Biacore^®^, 30 min).

### 3.3. Characterization Methods

In order to prove the chemical functionalization of the GaAs samples by the mixture of thiolates, we performed fluorescence characterization of the surface, using an NHS-modified probe, Cyanin5-mono NHS ester (from Amersham Biosciences), which is able to bind specifically to COOH chemical groups present on the functionalized GaAs surface. Using this probe, we sought to prove: 1) the presence of COOH groups on the GaAs surface after functionalization, and 2) that specific binding of the protein on the functionalized GaAs surface occurs after the EDC-NHS activation step of the thiolate layer. For this characterization, we used a Confocal fluorescence sensor (Fluo Sens DD, from ESE Embedded Systems Engineering, GmbH, Germany) that enables scanning of the sample at a desired and constant distance, while registering the fluorescence emitted by the sample at 680 nm. This sensor was used in E2D2 mode, which means with excitation and emission wavelengths of 625 and 680 nm respectively. To achieve this characterization, after deoxidation and thiolate functionalization of the GaAs sample (either by 10% MHDA/90% MUDO or by 0% MHDA/100% MUDO), we incubated Cy5-NHS (at 10 µg/mL, in distillated water) for 30 min, rinsed extensively with water, then measured the fluorescence intensity of the surface. This approach gives a graph in arbitrary units (A.U.) corresponding to the intensity of fluorescence emission at 680 nm, as a function of the scanned sample area in cm.

An original combination of characterization methods, namely atomic force microscopy (AFM) and MALDI-TOF mass spectrometry (MS), was used to highlight respectively topographical modifications after protein immobilization, and to prove the potential for* in situ* protein detection on the GaAs surface. The AFM is a Veeco^®^ Multimode 2 with a nanoscope IIIa controller, used in contact-air mode. The Veeco^®^ silicon nitride NPS10 tips and appropriate scan settings were used to obtain the best scanning conditions for each test and were kept constant from one sample to another. Cantilever stiffness was adapted: we used an NPS10-D tip (low stiffness, *k* = 0.06 N/m) for classical imaging, a NPS10-B (k = 0.12 N/m) for medium scraping and a NPS10-A (greatest stiffness, *k* = 0.58 N/m) for strong scraping. In this paper, we present only images of 3 µm width, but we also performed smaller and larger scans to respectively obtain better resolution and verify the homogeneity of the layer on a large scale. Measurements were repeated in several places in order to validate images for each surface. The MALDI-TOF mass spectrometer is a Bruker Daltonics^®^ UltrafleXtreme. A home-made MALDI target was used to analyze samples in the chamber. The chips were processed with the automatic imageprep standard protocol. This procedure includes three steps: TCEP reduction (Tris(2-carboxyethylphosphine) 20 mM in NH_4_HCO_3_ buffer, 10 min at 37 °C), trypsin digestion (Trypsin Gold Mass Spectrometry Grade, Promega^®^, 10 ng/µL, 37 °C) and HCCA matrix deposition (α-cyano-4-hydroxycinnamic acid, Bruker Daltonics^®^, 1.5 mg/mL in 50/50 acetonitrile/TFA 0.25%). The automatic mode, FlexImaging, was used with constant parameters (number of shots 2000, random walk 20 µm and pitch 120 µm) to allow comparison of each sample. The data from mass spectrometry images were exported and treated with home-made software. Additional tandem mass spectrometry (MS^2^) analysis, consisting in a second fragmentation of one specific peptide (here, the 1960 Da peptide) was performed to identify the protein unambiguously. The research for identification was performed with Mascot Matrix Science in the SwissProt database. Table 2 summarizes the different chemical interfaces tested on GaAs surfaces with or without RSA grafting.

**Table 2:** Different chemical interfaces tested on the GaAs surface with or without RSA grafting.

N	Thiol SAM	RSA	Characterization methods
1	without	without	AFM
2	10% MHDA/90% MUDO	without	
3	without	with	AFM + MS/MS^2^
4	0% MHDA/100% MUDO	with	
5	3% MHDA/97% MUDO	with	
6	10% MHDA/90% MUDO	with	
7	20% MHDA/80% MUDO	with	
8	50% MHDA/50% MUDO	with	
9	100% MHDA/0% MUDO	with	

## 4. Conclusions

RSA protein grafting on (100) Gallium Arsenide substrate functionalized by a mixture of thiols was carried out and proved using an original combination of methods, namely atomic force microscopy and mass spectrometry. These characterization methods provided complementary information about the binding type and composition of the protein layer according to the chemical interface. Moreover, through fluorescence characterization of the functionalized surface, we proved that MHDA thiolates were present and reactive on the GaAs surface, since they were able to specifically immobilize NHS ester molecules. In addition, MS investigation of the protein layer proved the ability to analyze* in situ* the protein present on the surface. The 10% MHDA chemical interface creates specific covalent bonds between protein and the GaAs (100) surface, and presents an appropriate number of docking sites for grafting of a dense protein layer. Coupled with recent advances in the development of the resonant piezoelectric sensor and in the conception of a specific test bench, these results on biointerface optimization will enable us to propose a selective and efficient biosensor, presenting an optimized and highly controlled biointerface.
